# Community based yoga classes for type 2 diabetes: an exploratory randomised controlled trial

**DOI:** 10.1186/1472-6963-9-33

**Published:** 2009-02-19

**Authors:** Lana Skoro-Kondza, Sharon See Tai, Ramona Gadelrab, Desanka Drincevic, Trisha Greenhalgh

**Affiliations:** 1 Research Department of Primary Care and Population Health, University College London, London, N19 5LW, UK

## Abstract

**Background:**

Yoga is a popular therapy for diabetes but its efficacy is contested. The aim of this study was to explore the feasibility of researching community based yoga classes in Type 2 diabetes with a view to informing the design of a definitive, multi-centre trial

**Methods:**

The study design was an exploratory randomised controlled trial with in-depth process evaluation. The setting was two multi-ethnic boroughs in London, UK; one with average and one with low mean socio-economic deprivation score. Classes were held at a sports centre or GP surgery. Participants were 59 people with Type 2 diabetes not taking insulin, recruited from general practice lists or opportunistically by general practice staff. The intervention group were offered 12 weeks of a twice-weekly 90-minute yoga class; the control group was a waiting list for the yoga classes. Both groups received advice and leaflets on healthy lifestyle and were encouraged to exercise.

Primary outcome measure was HbA1c. Secondary outcome measures included attendance, weight, waist circumference, lipid levels, blood pressure, UKPDS cardiovascular risk score, diabetes-related quality of life (ADDQoL), and self-efficacy. Process measures were attendance at yoga sessions, self-reported frequency of practice between taught sessions, and qualitative data (interviews with patients and therapists, ethnographic observation of the yoga classes, and analysis of documents including minutes of meetings, correspondence, and exercise plans).

**Results:**

Despite broad inclusion criteria, around two-thirds of the patients on GP diabetic registers proved ineligible, and 90% of the remainder declined to participate. Mean age of participants was 60 +/- 10 years. Attendance at yoga classes was around 50%. Nobody did the exercises regularly at home. Yoga teachers felt that most participants were unsuitable for 'standard' yoga exercises because of limited flexibility, lack of basic fitness, co-morbidity, and lack of confidence. There was a small fall in HbA1c in the yoga group which was not statistically significant and which was not sustained six months later, and no significant change in other outcome measures.

**Conclusion:**

The benefits of yoga in type 2 diabetes suggested in some previous studies were not confirmed. Possible explanations (apart from lack of efficacy) include recruitment challenges; practical and motivational barriers to class attendance; physical and motivational barriers to engaging in the exercises; inadequate intensity and/or duration of yoga intervention; and insufficient personalisation of exercises to individual needs. All these factors should be considered when designing future trials.

**Trial registration:**

National Research Register (1410) and Current Controlled Trials (ISRCTN63637211).

## Background

Type 2 diabetes has reached epidemic levels and its incidence and costs continue to rise. Physical activity is an effective intervention, but few patients engage in it regularly (see discussion). Yoga is popular, has many hypothetical benefits, and may be a more acceptable than sporting activity for some sectors of the population.

Yoga and diabetes

'Yoga' as an intervention varies widely. While many researchers conceptualize yoga as a form of physical activity, others argue that yoga is a holistic intervention incorporating body postures (asanas), breathing techniques (pranayamas), meditation, cleansing, nutrition, modification of attitudes and behaviour, and mental discipline. [1]

The advantages of yoga as option for physical activity in diabetes include (a) the holistic philosophy in which physical exercises are linked to a wider a lifestyle package that also includes diet, relaxation and what western practitioners would call stress management; (b) low cardiovascular demands relative to other forms of exercise; (c) low impact, hence meets a need for people who are obese, have difficulties in mobilisation, or contra-indications (e.g. proliferative retinopathy) to strenuous exercise; and (d) it provides an alternative identity option (yoga practitioners do not see themselves as "sporty").

Five previous randomised trials have been published of yoga in Type 2 diabetes. [2-6] The only UK trial recruited 21 participants from a hospital clinic and showed a highly significant fall in mean HbA1c (1.6%) in the yoga group over a three-month intervention period. [3] A US study based in primary care randomised 60 patients and showed no significant impact of yoga overall, though a subgroup of patients with high initial HbA1c levels showed a significant fall. [6] Three trials from India, in which a total of 310 patients were randomised, all showed a positive but statistically non-significant impact of yoga on glycaemic control. [2,4,5] Two recent systematic reviews concluded that current evidence suggests (but does not prove) that yoga may be efficacious in Type 2 diabetes; that publication bias may have occurred; and that an adequately powered, well-designed randomised controlled trial is needed to remedy the methodological deficiencies of previous studies. [7,8]

Yoga can be thought of as a 'complex intervention', for which the Medical Research Council recommends an extensive development period to achieve a robust, theory-based mechanism of action (phase 1), optimise the coordination and delivery of components (phase 2), and test these in exploratory trials (phase 3) before undertaking a definitive randomised trial (phase 4). [9] This exploratory trial was designed as a 'phase 3' study in the development of the intervention. As such, the trial was intentionally underpowered, since the purpose of the study was not to obtain a definitive effect size but to optimise processes and address the fidelity of the intervention in a real-life setting.

## Methods

### Project management and governance

Research Ethics Approval was granted by Barnet, Enfield and Haringey Local Research Ethics Committee, REC Number 04/Q0509/32. The trial was prospectively registered on the National Research Register (ID 1410) and Current Controlled Trials (ISRCTN63637211). A steering group with representation from academics, local GP surgeries, yoga therapists, and a statistician met regularly through the study, considered emerging data, and approved ongoing modifications to the protocol.

### Setting

The study was conducted in two north London boroughs. Borough A was in the lowest quartile for socio-economic deprivation, and 55% of the population were from ethnic groups other than white British. Borough B was more affluent, and its ethnic mix was close to the London average (40% from non-white British groups). Yoga classes at Borough A were held at a local leisure centre and at Borough B in a meeting room at one of the general practice surgeries.

### Study design

**Figure 1:**
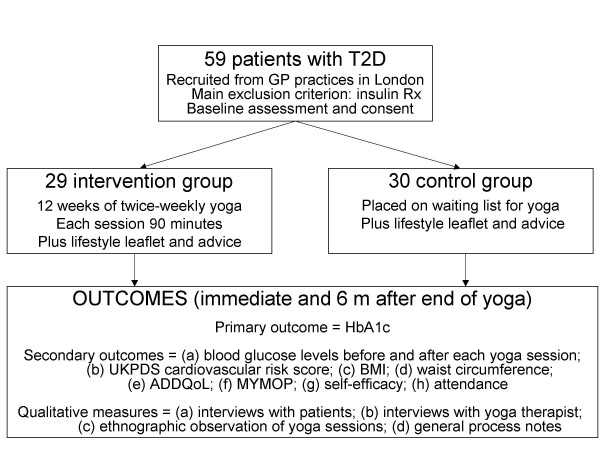
**Summary of study design**.

Exploratory randomised controlled trial with in-depth process evaluation, informed by the Medical Research Council framework. [9] The study was designed to generate data on (a) how best to recruit GP practices and patients; (b) operational aspects of delivering the yoga (e.g. where, when and how often); (c) what sort of yoga exercises to would be acceptable and efficacious; (d) how to maximise attendance and acceptability of the classes; (e) patients' and therapists' qualitative experiences; (f) the feasibility of different outcome measures; and (g) an estimate of effect size to inform the sample size calculation in a definitive trial. The design is summarised in Figure 1.

### Sampling and recruitment

In Borough A, we wrote to 60 general practices inviting them to participate. Those showing interest were visited and a standard information pack given. Participating practices agreed to recruit eligible patients opportunistically; posters were provided to put up in these surgeries. Because of low recruitment using this method, we adopted a more pro-active approach in Borough B. We approached two general practice surgeries whom we knew to be interested in both diabetes and research. We obtained a practice list of diabetic patients and drafted an invitation letter on practice headed notepaper for the general practitioner to send to potentially eligible patients. We followed up non-responders in Borough B by letter and telephone call.

### Inclusion and exclusion criteria

We included all diabetic patients over 18 with diabetes not treated with insulin. We excluded insulin treated patients because precipitous falls in blood glucose levels may occur during yoga in patients on insulin. [10] Exclusion criteria were contra-indication to physical exercise (e.g. unstable or under-investigated coronary heart disease, disseminated cancer, severe osteoporosis), inability to join in a yoga class (e.g. a relevant mental illness), or inability to understand English sufficiently to participate in the class (major cognitive deficit, poor English fluency).

### Assessment and randomisation

Interested patients were invited to an assessment visit at their general practice or a local surgery, at which the study was explained and informed consent obtained before performing a baseline physical examination and ordering blood tests. We also recorded self-assessed ethnicity and Index of Multiple Deprivation of the person's home postcode (derived from national census data and scored from 0.6 to 86 with a national mean of 21 and median of 17, in which a higher score means greater deprivation[11]). Eligible participants were randomised using opaque sealed envelopes to either yoga or a waiting list control arm; all participants eventually had 12 weeks of yoga classes. Because of the small numbers a block randomisation design was used to ensure equal numbers in each group.

### Intervention

The intervention group was encouraged to attend a total of 24 90-minute yoga classes over 12 weeks. Classes were run by an experienced yoga teacher who used judgement to adapt the exercises to the needs and abilities of the participants. The exercises focused primarily on breathing and relaxation (pranayama) but also included gentle stretching and postures (asanas). All participants in the yoga group were given their own yoga mat and belt, a leaflet of yoga exercises, and an audiotape by a yoga therapist; the waiting list control participants also received these after the end of the study period. In addition, both intervention and control groups were given a leaflet about exercise in diabetes and encouraged to take regular exercise.

### Outcome measures

The primary outcome measure was HbA1c. Secondary outcome measures (predefined before the study began) were UKPDS cardiovascular risk score (a composite of blood pressure, smoking status, lipid ratio, presence of atrial fibrillation, and HbA1c which has high predictive value in diabetes),[12] and three candidate psychometric scales: quality of life (using the diabetes-specific ADDQoL instrument[13]), self-efficacy (using a diabetes-specific adaptation[14]), and MYMOP (Measure Yourself Medical Outcome Profile[15]). These measures were taken at baseline, immediately on completion of the yoga class (or control period), and six months later. In addition, we monitored fingerprick blood glucose levels before and after each yoga class.

### Process measures

We held two focus groups with each group of participants – one before the start of the yoga class and one on the day of the last class. In the first of these we explored their hopes, expectations and concerns about the yoga class. In the second, we asked open-ended questions about the class (e.g. "how did it go?" "what do you think you got out of it?" "were there any problems?") and whether expectations had been met.

Because of low attendance at the Borough A class, we decided to introduce prompting for the Borough B class. With consent, a telephone or text reminder was sent the day before the class. For those who had chosen to be contacted by text, we sent another text the day after the class asking "how was the class?". Text responses were included in our qualitative data set. In addition, ten of the 29 participants randomised to yoga consented to having an individual semi-structured interview, at which we asked similar questions to those in the focus group (but with the opportunity for a private reply), and also asked specifically about factors affecting their attendance at the classes and ability to follow the exercises set.

Members of the research team (LS-K and RG) attended a total of 10 yoga classes and wrote up brief ethnographic field notes after each class.

We conducted a semi-structured interview with each yoga teacher towards the end of the series of classes, in which we encouraged them to comment on the motivation and ability of the participants; which exercises had been given and why; perceptions of barriers to effective participation, and what they would advise us when designing a further trial.

The data sources for the process analysis are summarised in Table 1.

**Table 1:** Data sources for process analysis

**Source of data**	**Type and quantity of data**	**Insights from these data**
Yoga participants	Attendance records for all classes 4 focus groups (2 before class began, 2 after last class) 10 semi-structured interviews Text messages from 7 participants	Whether people attended and if not, why not Hopes and fears for yoga Evaluation of their own performance and perceptions of barriers to yoga exercise Confidence and motivation to continue

Yoga teachers	2 semi-structured interviews (one with each teacher)	Type of individual who might benefit Type of exercise that might be beneficial Suggestions for intensity and duration of exercise sessions

Ethnographic observation	Field notes from researchers' attendance as participant observers at yoga class	Participants' engagement with the class Practical constraints e.g. noise, room size

Documentary sources	Letters, emails, and file notes relating to setting up and running the project (e.g. relating to gaining access to practices, recruitment, linking with clinical care)	Operational challenges and how they might be reduced or avoided next time

### Data processing and analysis

All quantitative analyses were conducted in SPSS Version 12 using paired sample t tests to compare paired measures (physiological and biochemical measures, questionnaire scores, pairing before and after measures in each participant) and analysis of covariance to compare trend over time in the following variables (all of which were approximately normally distributed in the sample): HbA1c, body mass index, waist-hip ratio, systolic and diastolic blood pressure, lipid levels (total cholesterol, LDL, HDL, triglycerides), UKPDS risk score, and scores on the psychometric instruments (self efficacy, MYMOP and ADDQOL).

Interview and focus group data were transcribed and analysed for themes using the constant comparative method (i.e. as qualitative data were generated, themes in the new data were compared with those already identified from older data, thus iteratively refining the themes). [16] This process was aided by the use of an Excel spreadsheet for framework analysis,[17] and by regular discussions between researchers.

## Results

### Recruitment

Recruitment was difficult and slow. Of 60 general practices approached in Borough A, only 12 were interested and nine of these recruited very few participants. In Borough B, where we used practice diabetes registers, recruitment was much quicker but most patients on the register proved ineligible (because of insulin treatment, ischaemic heart disease, cerebrovascular disease, or other co-morbidity). In one practice, for example, only 53 out of 187 adult patients on the register were eligible and only six of these (3% of those the register) agreed to participate. Reasons given for declining included not interested (most commonly), too busy, not keen on the research aspect of the study, anxieties about their ability to do yoga ("not bendy enough"), and already attending a yoga class (two patients).

### Participants

The 59 participants comprised 13 men and 36 women. Mean age was 60 (SD 10) years, and mean duration of diabetes 30 (SD 5) years. Their self-assessed ethnicity was white British (10); other European or Mediterranean (4); African (14); and Asian (31). The mean deprivation score was 20.5, median 28.3, and range 4.2 to 60.0, indicating a wide range of socio-economic backgrounds but skewed towards the more deprived sectors.

### Attendance

Overall, attendance at the classes was 50%, with the number of classes attended ranging from zero to 24. The commonest reason given for not attending was that the class was at an inconvenient time (especially for those at work or studying) or conflicted with another appointment (e.g. dentist, picking up a child). Text and phone reminders did not substantially increase attendance, mainly because very few people gave forgetting as the usual reason for non-attendance, though a minority of participants found the reminders helpful.

### Observation of the yoga groups

Participants appeared to engage with and enjoy the yoga classes, where much of the time was spent on relaxation exercises. One of the yoga teachers spent considerable time 'tailoring' exercises for each participant so that everyone could join in somehow (e.g. if a person could not lie on the floor, a chair-based alternative was negotiated). Several participants in the other teacher's group spent parts of the class not participating at all. Pre-and post-yoga fingerprick blood glucose tests showed that 80% of participants experienced a fall in their blood glucose level during the class (mean change -2.1 mmol/L; SD 1.9 mmol/L; p < 0.0001), and one in 20 participants had a dramatic drop of 5 mmol/L or more.

### Participants' perspective

All participants had high hopes that yoga would improve their general well-being and help control their diabetes. All were nervous before the course that they would not cope with the classes, and some expected to be asked to undertake complex contortions. They enjoyed the classes (which they found much gentler than they had feared), spoke highly of the teacher, and said they felt much better after a class. This was true both for high and low attenders. Participants said found it much easier to do yoga in a group than alone. They valued the social aspects of the group, and some expressed disappointment that there was no opportunity for socialising immediately after the class (because the venue had been booked for a limited time).

### Yoga teachers' perspective

Yoga teachers felt that participants benefited from the classes, but that very few would have been suited to a 'general' yoga class because of frailty, stiffness, lack of fitness, lack of confidence, or co-morbidity. Both teachers felt that all participants should have a personalised plan of yoga exercise which took into account their physical fitness, co-morbidity and confidence. They also felt that sessions should be longer than an hour (whilst the venue had been booked for 90 minutes, only 60 minutes was spent exercising), and that the course should last at least six months. They were unhappy with the venues provided, which were perceived to be too noisy and not conducive to the spiritual aspects of yoga (for example, the sports centre venue was characterised by repeated intercom announcements). Teachers were surprised that participants had not undertaken any yoga exercises at home and felt that in future research, agreement to practise regularly at home should be a precondition for inclusion in the trial and this aspect of the intervention should be given more emphasis in class.

### Impact of the yoga intervention

On an intention to treat basis, there was no difference in any outcome measure between the two groups. In the intervention group, HbA1c fell slightly from 7.06 to 6.86 immediately after the course (mean change -0.02; 95% CI -0.40–0.001), but rose again to 7.01 six months later (mean change -0.05; 95% CI -0.26–0.16). The corresponding figures for the control group were 7.03 to 6.95 (mean change -0.07; 95% CI -0.21–0.007) and 7.30 six months later (mean change -0.28; 95% CI -0.10–0.66 95%CI). None of these differences was statistically significant. No other outcome measure showed a significant change. In terms of psychometric scales, the ADDQoL proved easy to administer and produced reliable data (though no changes were observed in score), but the diabetes self-efficacy scale was difficult to complete and analyse, and the MYMOP scale was very unreliable (because participants' goals were not stable over time).

## Discussion

This exploratory trial, which failed to demonstrate a significant impact of yoga in Type 2 diabetes, suggests that recent reports about the benefit of this intervention may have been premature. [1,10] The negative finding of this study may be explained either by lack of efficacy (yoga has no effect on glycaemic control or other cardiovascular risk factors) or by a number of factors that combined to attenuate the impact of a potentially effective intervention. In a future, larger study, a prospective per-protocol analysis (in addition to the main intention-to-treat analysis) might help to adjudicate between lack of efficacy of the intervention and poor attendance.

Our process data suggest that factors which could have contributed to a possible under-estimate of the efficacy of the intervention may have included good background control in the population targeted (the mean HbA1c of the adult diabetic population in participating practices was 6.9 mmol/L and few patients with poor glycaemic control entered the study); practical and motivational barriers to class attendance; physical and motivational barriers to engaging in the exercises; inadequate intensity and/or duration of yoga intervention; and insufficient personalisation of exercises to individual needs.

The trade-off between 'personalising' exercises and delivering a 'standardised' intervention raises questions about fidelity of intervention which should also be considered in future trials, perhaps with attention to Hawe's writing on achieving a theoretically coherent 'core' intervention but applying flexibility to individual needs. [18]

A striking finding in our qualitative data was the mismatch between what people said about the yoga classes (enjoyable, make me feel better, improves my diabetic control) and their lack of commitment to attending them or continuing the exercises at home. It would appear that whilst participants *valued *the yoga class, they did not *prioritise *it, and some only attended if nothing else came up in the time slot. In this respect, attending the yoga class was rather like 'going to the gym' and did not have the same medical significance for participants as (say) an appointment with a health professional. Whilst 'community based' yoga may have a more holistic ethos, a previous randomised trial showing dramatic impact of yoga on HbA1c (in which attendance was very high) was held in a hospital clinic and the yoga course "prescribed" by the patient's diabetologist (Monro R, personal communication). [3]

Changing one's lifestyle, especially from sedentary to physically active, is difficult. Compliance with exercise regimens in diabetes is notoriously low, [19-22] especially in 'free living' community settings,[21,23,24] extremes of age, [25-28] and minority ethnic groups. [29-31] Exercise programmes offered in research studies are rarely sustained beyond the intervention phase. [21,22] Barriers to taking regular exercise in diabetes include low motivation[19,21] a personal identity that does not embrace athleticism,[32,33] and lack of social capital and social support. [34] Whether yoga should be 'medicalised' (e.g. as a 'prescription for exercise' intervention) to increase the priority given to it by patients is not an easy question to answer. Whilst this move may improve attendance, it also sits oddly with the ethos of a 'holistic' intervention intended to transform mind, body and spirit.

A recent review article by Alexander et al considered the uptake of yoga in diabetes from the perspective of the social determinants of health. [1] Promoting behavioural interventions such as physical activity for diabetes, they argue, implicitly places responsibility (and, where relevant, blame) on the individual, and diverts attention from the economic, social and cultural barriers to partaking in such activity. Participation in yoga has a very strong class, ethnicity and age bias (like other complementary and alternative medicine interventions, it is undertaken predominantly by the relatively young, educated, white middle classes, despite its origins in the East). [1] Our finding that most people with Type 2 diabetes who are eligible and willing to attend yoga classes in our London setting are from non-white ethnic groups, lower socio-economic backgrounds, and close to or above retirement age suggests that a key component of a future trial should probably be 'marketing' the idea of yoga to a group who might not otherwise be drawn to this activity.

## Conclusion

Taking into account the process data generated by this study, we make the following recommendations for the design of future trials:

### Recruitment

• Must be simple and involve no additional work for general practitioners or their staff

• Greatest interest is likely to come from the recently retired (60–69) as they have time to attend and may be well motivated

### Venue

• Must be 'fit for purpose' – ideally a quiet centre where yoga classes are already being held

• Must provide 'social' incentive e.g. meeting space to chat, lunch after the session

### Maximising attendance

• Pay careful attention to convenience of sessions

• Text or phone reminders may help some participants but most non-attendance is not due to forgetting

• Use yoga teacher to increase motivation

### The exercises

• Each participant should have a 'personalised' package tailored to their individual needs and motivation

• Strenuous physical exercises or those needing flexibility are unsuitable for most diabetic people

• Monitor blood glucose levels closely as precipitous drop may occasionally occur even in those not taking insulin

### How often should yoga be done?

• Aim should be several times a week for at least 90 minutes each time, but because of problems with attendance, most of this must be done at home

• Participants must agree to practice at home as a precondition for inclusion*

• Teachers must motivate people to exercise at home and give specific 'homework'

• Home 'yoga kit' (e.g. mat, belt, block) seems to be valued but supplying it will not automatically lead to yoga being done at home

### Process/outcome measures

• It is very important to measure attendance

• HbA1c and other blood tests are feasible but should be linked in with people's routine diabetes checks to avoid over-investigating

• ADDQoL appears a robust and acceptable measure (but may not be sufficiently sensitive to change)

• Further ethnographic observation would provide additional insights on what sort of yoga exercises are helpful for what sort of patient

## Competing interests

The authors had no competing interests. The sponsor of this study, Novo Nordisk Research Foundation, to whom we are grateful for financial support, had no role in the study design, data collection, data analysis, data interpretation, or writing of the report. The corresponding author had full access to all data in the study and had final responsibility for the decision to submit for publication.

## Authors' contributions

LS-K conceptualised the study, led the recruitment of practices and patients, undertook assessment and randomisation of participants, and led collection of data. RG and DD assisted with assessments and data collection, and RG led the sub-study of text and phone prompting. SST led the quantitative analysis. TG provided methodological support and led the analysis of qualitative data with assistance from RG and LS-K. TG and LS-K wrote the paper. All authors read and approved the final manuscript.

## Pre-publication history

The pre-publication history for this paper can be accessed here:


